# INVESTIGATING THE NEURAL CORRELATES OF SOCIAL AND INDIVIDUAL SINGING IN PERSONS WITH DEMENTIA

**DOI:** 10.1093/geroni/igad104.2549

**Published:** 2023-12-21

**Authors:** Nicholas Tamburri, Debra Sheets, Andre Smith, Stuart MacDonald

**Affiliations:** University of Victoria, Victoria, British Columbia, Canada; University of Victoria, Victoria, British Columbia, Canada; University of Victoria, Victoria, British Columbia, Canada; University of Victoria, Victoria, British Columbia, Canada

## Abstract

Music interventions for persons with Alzheimer’s Disease and related dementias (PwADRD) have documented psychological benefits; however, the neurological correlates underlying these advantages remain less certain. Using functional near infrared spectroscopy (fNIRS), the present study i) investigated within-person patterns of cortical oxygenation during choral vs. individual singing; ii) explored how singing context (choral vs. individual) modulated patterns of functional connectivity (FC) within and across frontal and parietal cortices; and iii) leveraged these functional activations as predictors of cognitive status (degree of impairment) in a series of discriminant function analyses (DFA). Participants were 13 PwADRD who volunteered from a larger, ongoing social-cognitive choir intervention. fNIRS data were collected using a TechEn Cw6 system during both choral and individual singing conditions. Paired sample t-tests evaluated differences in activation patterns between singing conditions, with DFA used to determine whether these activations and neuropsychological function were predictive of between-person differences in cognitive impairment. Significant differences in cerebral oxygenation were identified in the right anterior PFC, with individual singing associated with significantly greater cortical oxygenation relative to group singing. Although not significant, individual singing was also associated with bilateral increases in cortical oxygenation across the majority of fNIRS channels, as well as increased FC, relative to group singing. The DFA analyses were not significant. This novel study is the first to examine differences in music-cognition correlates across environmental contexts for PwADRD. Patterns of functional activation suggest that choral singing in particular may represent an optimal lifestyle activity, placing comparatively fewer demands on cognitive function.

